# Lipid storage changes in human skeletal muscle during detraining

**DOI:** 10.3389/fphys.2015.00309

**Published:** 2015-11-03

**Authors:** Rong Zhu, Caiyun Wen, Jiance Li, M. Brennan Harris, Yung-Yang Liu, Chia-Hua Kuo

**Affiliations:** ^1^School of Sports Science, Wenzhou Medical UniversityWenzhou, China; ^2^Department of Radiology, The First Affiliated Hospital of Wenzhou Medical UniversityWenzhou, China; ^3^Department of Kinesiology and Health, College of William and MaryWilliamsburg, USA; ^4^Institute of Clinical Medicine, School of Medicine, National Yang-Ming UniversityTaipei, Taiwan; ^5^The Chest Department, Taipei Veterans General HospitalTaipei, Taiwan; ^6^Laboratory of Exercise Biochemistry, Department of Sports Sciences, University of TaipeiTaipei, Taiwan

**Keywords:** muscle hypertrophy, strength, fatty infiltration, training, creatine, EMCL, IMCL, exercise

## Abstract

Exercise training is known to increase intramuscular triglyceride content in both trained and untrained legs. The purpose of the study was to determine the changes of intramyocellular lipids (IMCL) and extramyocellular lipids (EMCL) of both trained and untrained legs during detraining. We measured both IMCL and EMCL levels in previously trained vs. untrained legs during 4-weeks of detraining after 6-weeks of strength training. Eight young men (aged 21.4 ± 1.4 years) trained their vastus lateralis muscle in one leg using a dynamometer, whereas the contralateral leg served as untrained control. Muscle cross-sectional area (CSA), IMCL, EMCL, total creatine (creatine + phophocreatine) of extensor (vastus lateralis) muscles were assessed using magnetic resonance imaging (MRI) and proton magnetic resonance spectra (^1^H-MRS) before training, 3 days after and 28 days after the last bout of training. CSA was increased in both legs by Day 3 after training, and was still high at Day 28 post-training; IMCL increased in both legs by Day 3 after training, then decreased at Day 28 post-training only in the untrained leg; EMCL shows no significant change by Day 3 after training, but at Day 28 post-training has increased in the trained leg and decreased in the untrained leg; total creatine did not change significantly.

**Conclusion:** Decreases of IMCL and EMCL storages in previously untrained leg during detraining indicates an ectopic influence on tissue lipid storage by different metabolic demand among tissues in the same human body.

## Introduction

Lipid, as one of the most abundant sources of stored reducing hydrocarbon in the human body, not only serves as a fuel for ATP production, but also the main source of acetyl-CoA and as a source of hydrocarbon backbone for the syntheses of diverse biological compounds, such as phospholipid, cholesterol, ketones, and some amino acids for the construction of new cells. In humans, skeletal muscle is the major consumer of lipids during post-absorptive and fasting conditions (Coppack et al., 1990). Muscle hypertrophy after exercise training and atrophy after disuse reflect the exceptional adaptability of skeletal muscle to relocate hydrocarbon sources in response to changing physical demand.

Lipids in skeletal muscle can be divided into two compartments: intramyocellular lipids (IMCL) and extramyocellular lipids (EMCL), by proton magnetic resonance spectroscopy (^1^H-MRS), which has been successfully used to quantify the lipid content of skeletal muscle for two decades (Steidle et al., 2002; Godoy-Matos et al., 2010; Redzic et al., 2014). The methylene protons (-CH_2_-)_n_ of IMCL and EMCL dominate the proton (^1^H) magnetic resonance spectra of lipids in skeletal muscle. The methodology of using ^1^H-MRS to separate IMCL and EMCL has been justified in skeletal muscle by showing a severely asymmetrical distribution (Steidle et al., 2002) and the intervention that changes lipid distribution (Godoy-Matos et al., 2010). During an acute bout of moderate exercise, IMCL content, but not EMCL, decreases significantly in skeletal muscles (Rico-Sanz et al., 2000; Schrauwen-Hinderling et al., 2003). Endurance training with sufficient period of recovery significantly increases intramuscular triglyceride content (Shaw et al., 2012) consistent with increased IMCL (Haus et al., 2011).

Lipid storage in a tissue may be influenced by metabolic demand of ectopic tissue. For example, IMCL content of non-exercising human skeletal muscle is increased after exercise in trained cyclists before and after a 3-h cycling in the exercising vastus lateralis muscle (Schrauwen-Hinderling et al., 2003). In transgenic animal experiments, it have shown a decrease size of adipose tissue by increasing muscle growth (McPherron and Lee, 2002; Diaz et al., 2012). It is currently unknown whether EMCL as a rich storage pool of lipid in a sedentary leg can be influenced by training activity of another leg. EMCL is located much closer to working muscle fibers than abdominal lipid. Extracellular space is an aqueous environment, thus EMCL of leg muscles detected by proton MRS mirrors the amount of adipocyte infiltration within skeletal muscle (Popadic Gacesa et al., 2011). Recently, an inverse relationship between EMCL and physical function has been described in a preliminary study using ^1^H-MRS (Redzic et al., 2014). Given the fact that physical function is altered by exercise training, the purpose of the study was to determine the changes of EMCL and ICML in both trained and untrained legs in response to cessation of training.

## Materials and methods

### Participants

No physical training was performed 2 days prior to the study. All participants were non-athletes and healthy according to annual medical examinations. Baseline characteristics of the participants are summarized in Table 1. Prior to the ^1^H-MRS study, informed written consent was obtained from all eight male participants (aged 21.4 ± 1.4 years). Institutional Review Board of Wenzhou Medical University approved the protocol. All procedures were conducted according to the principles expressed in the Declaration of Helsinki.

**Table 1:** **Physical characteristics**.

**Participants**	**Age (yr)**	**Height (cm)**	**Weight (kg)**	**BMI**
A	21	180	73	22.5
B	20	180	66	20.4
C	20	175	58	18.9
D	20	180	70	21.6
E	19	175	68	22.2
F	19	178	72	22.7
G	19	175	63	20.6
H	20	176	70	22.6

### Training protocol

To avoid possible individual variations among participants, all subjects were asked to perform one-leg exercise training on an Isomed 2000 dynamometer (D&R, Hemau, Germany) and their contralateral leg served as control. Participants were requested to conduct brief warm-up stretching lasting < 5 min without significant subjective exertion before training. They were then asked to train their quadriceps and hamstring muscle groups in one leg with their greatest endeavor. For the first 3 weeks, exercise intensity included isokinetic (concentric/concentric) maximal knee flexion and extension at 60°/s (six repetitions for six sets with a 1-min rest), and at 180°/s (15 repetitions for two sets with a 1-min rest) with a 1-min rest interval between different angular velocities, 3 days a week. For the last 3 weeks, exercise volume maintained at an angular speed 60°/s six times for six sets with a 1-min rest interval, but 180°/s for 15 times with a 1-min rest interval for three sets, 3 days a week.

Isokinetic strength measurements were conducted using the dynamometer in consistent to which used in training. Before the testing procedure, all the subjects performed conditioning exercises and stretching of the lower extremities to warm up for 5 min. The attachments of participant to the dynamometer were readjusted to the center of motion of the lever arm and aligned as accurately as possible with the slightly changing flexion-extension axis of the joint each time before testing. A restraining belt was placed obliquely over the shoulder and thigh to secure the position of participant during motion. The resistance pad was placed on the distal site of tibia. The range of motion of the knee joint was set at 0–90°. Bilateral isokinetic (concentric/concentric) maximal knee flexion and extension at the angular speed of 60°/s (five repetitions), 180°/s (15 repetitions) were accomplished. Between the two sessions, subjects rested for 1 min. Vocal encouragement during the tests was consistently standardized. Flexion and extension peak torque values automatically produced by the device.

### MRS

Participants sat at rest for more than 5 min in the scan room. The entire scanning session included data acquisition for 90 min or less and was tolerated by all subjects at pre-exercise, post-training (3 days after the last bout of training) and detraining (28 days after the last bout of training). MR Imaging and spectroscopy data were acquired on a 3.0-T Philips Achieva MRI scanner (Philips Healthcare, Best, Netherlands) by using a 16-channel torso coil. In each examination, subjects lay in a supine position with both legs placed along the axis of the coil and immobilized by firm padding. Transverse and sagittal longitudinal relaxation time-weighted MR images (repetition time minimum, echo time 15 ms) were acquired to determine the placement of the^1^H-MRS voxels, with a slice thickness of 5 mm, a 40-cm field of view, and 128 × 256 data matrix. Spectra were obtained from the vastus lateralis of both legs, using a point-resolved spectroscopy sequence with a repetition time-to-echo time ratio of 2000/50 ms and an 18-mm^3^ voxel positioned within each of the muscles examined. The number of signal averages was 96 and the spectral collection time was 3 min 48 s.

### Cross-sectional area (CSA) analysis

Anatomical CSA of the thigh muscles was determined at mid-femur by MRI. The femur length was defined from the most proximal prominence of the greater trochanter to the most distal border of the lateral femur condyle. CSA analysis of individual muscles of the thigh were determined by using the computer software of 3.0-T Philips Achieva MRI scanner (Philips Healthcare, Best, Netherlands) before training, 3 days after and 28 days after the last bout of training.

### Data analysis

**Figure 1:**
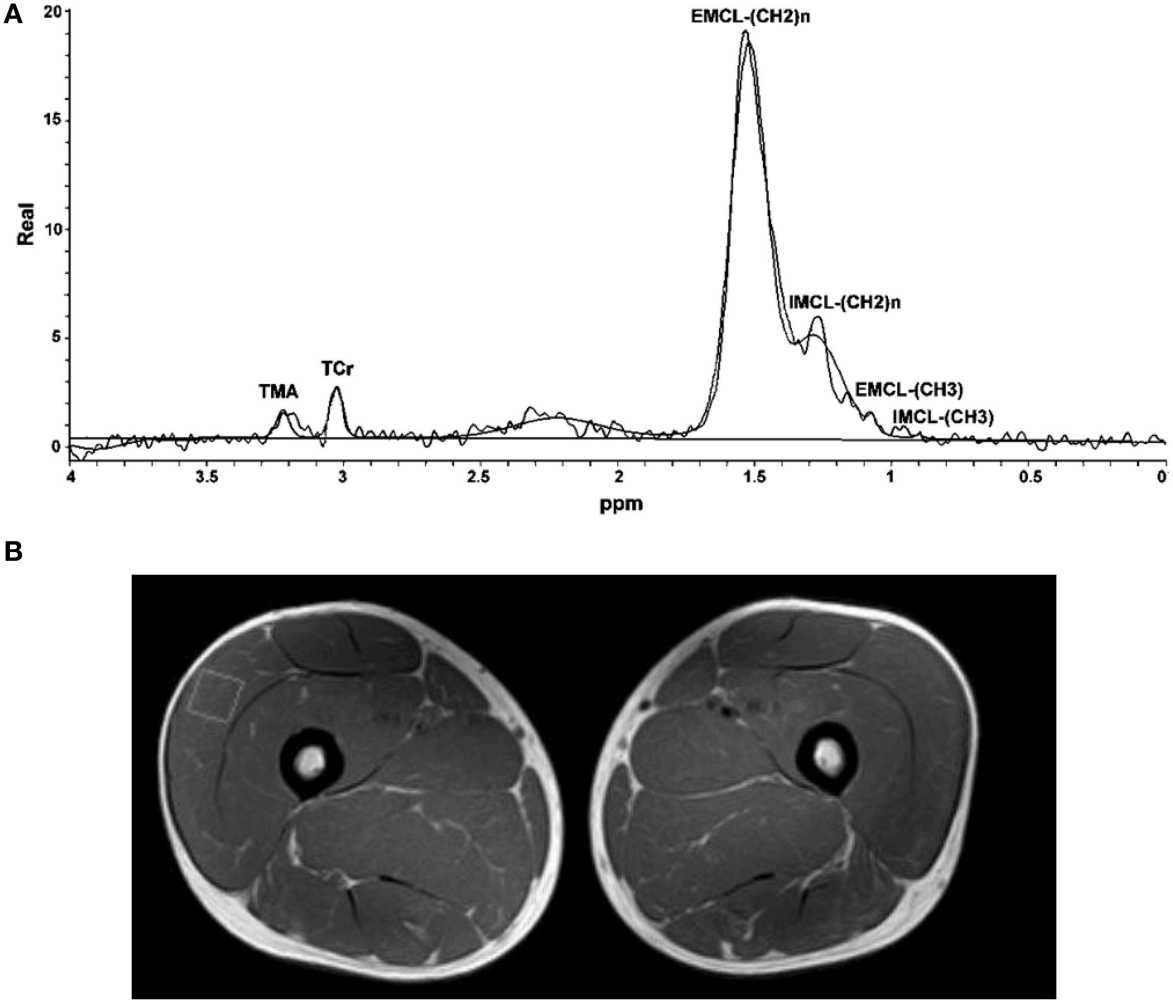
**^1^H-MRS spectra of skeletal muscle and fitted data (A)**. Placement of voxel for spectroscopy in vastus lateralis. Square in MR image shows the position of the ^1^H-MRS voxels of leg muscles examined **(B)**. EMCL, Extramyocellular lipid; IMCL, Intramyocellular lipid; Cr, Creatine (creatine + phosphocreatine).

The analyses of *in vivo* ^1^H-MRS data of thigh muscles by Spectroview Software from Philips Achieva 3.0T TX EWS were performed in the time domain directly on free induction decays (FIDs). Six resonances were described by six Gaussian line shapes in the frequency domain (Figure 1): *(a)* TMA (trimethylamine) peak at a resonance of 3.2 ppm, *(b)* TCr (total creatine), including free creatine (Cr) and phosphocreatine (PCr) peak at a resonance of 3.02 ppm, *(c)* EMCL-(CH_2_)_n_ [extramyocellular lipid-(CH_2_)_n_] peak at a resonance of 1.5 ppm and *(d)* IMCL-(CH_2_)_n_ [intramyocellular lipid(CH_2_)_n_] peak at a resonance of 1.3 ppm, *(e)* EMCL-(CH_3_) peak at a resonance of 1.1 ppm and *(f)* IMCL-(CH_3_) peak at a resonance of 0.9 ppm. The water signal peaks at 4.7 ppm. Unsuppressed water spectra obtained from the same voxel were used as the internal reference for the relative quantification of the metabolite resonances. All non-water resonances were removed from the unsuppressed FIDs by using the Hankel- Lanczos single-variable decomposition method (de Beer et al., 1992).

### Statistical analysis

Due to small sample size, the Shapiro-Wilk test was used to test whether the data were normally distributed, and Levene's test was used to confirm the homogeneity of variances. Paired data were analyzed using the non-parametric Wilcoxon's signed rank test. To analyze changes over time, a Friedman test was used and when a significant F-ratio was found, Wilcoxon's signed rank test was used for *post-hoc* analysis. Data are presented as % pre-trained level in means ± standard error (SE). A total of six subjects would have been required for 80% power, according to power estimation calculated to determine sample size. A *p* < 0.05 was considered significant for all tests.

## Results

**Figure 2:**
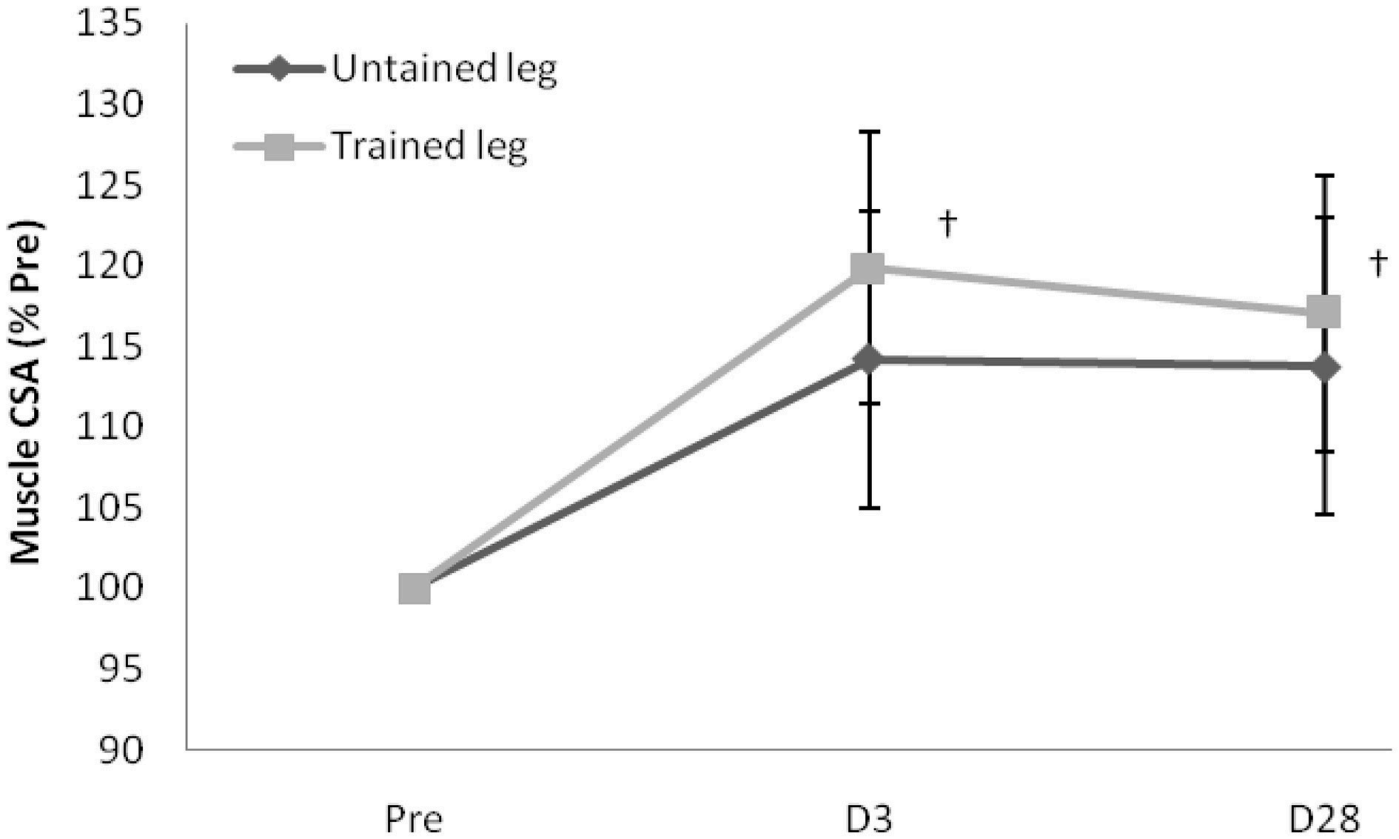
**Increased muscle cross-sectional area (CSA) of trained leg well-maintained during the 28-day detraining period**. Values are expressed as % of Pre. ^†^significance against Pre, *p* < 0.05. D3 and D28: Day 3 and Day 28 after the last training bout.

**Figure 3:**
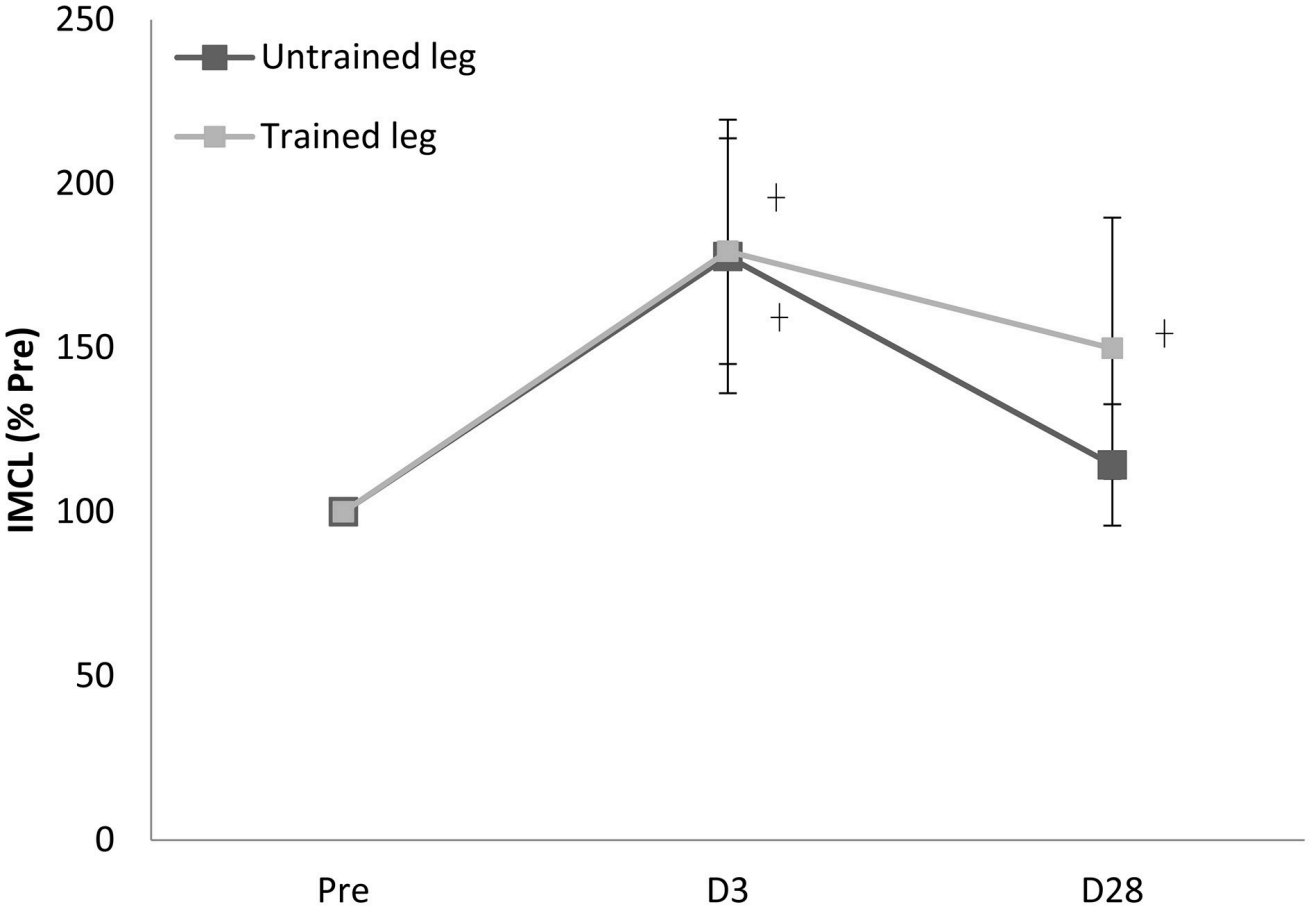
**Training effect on increasing intramyocellular lipid (IMCL) content of trained vastus lateralis vanished in 28 days after cessation of training**. ^†^significance against Pre, *p* < 0.05. D3 and D28: Day 3 and Day 28 after the last training bout.

**Figure 4:**
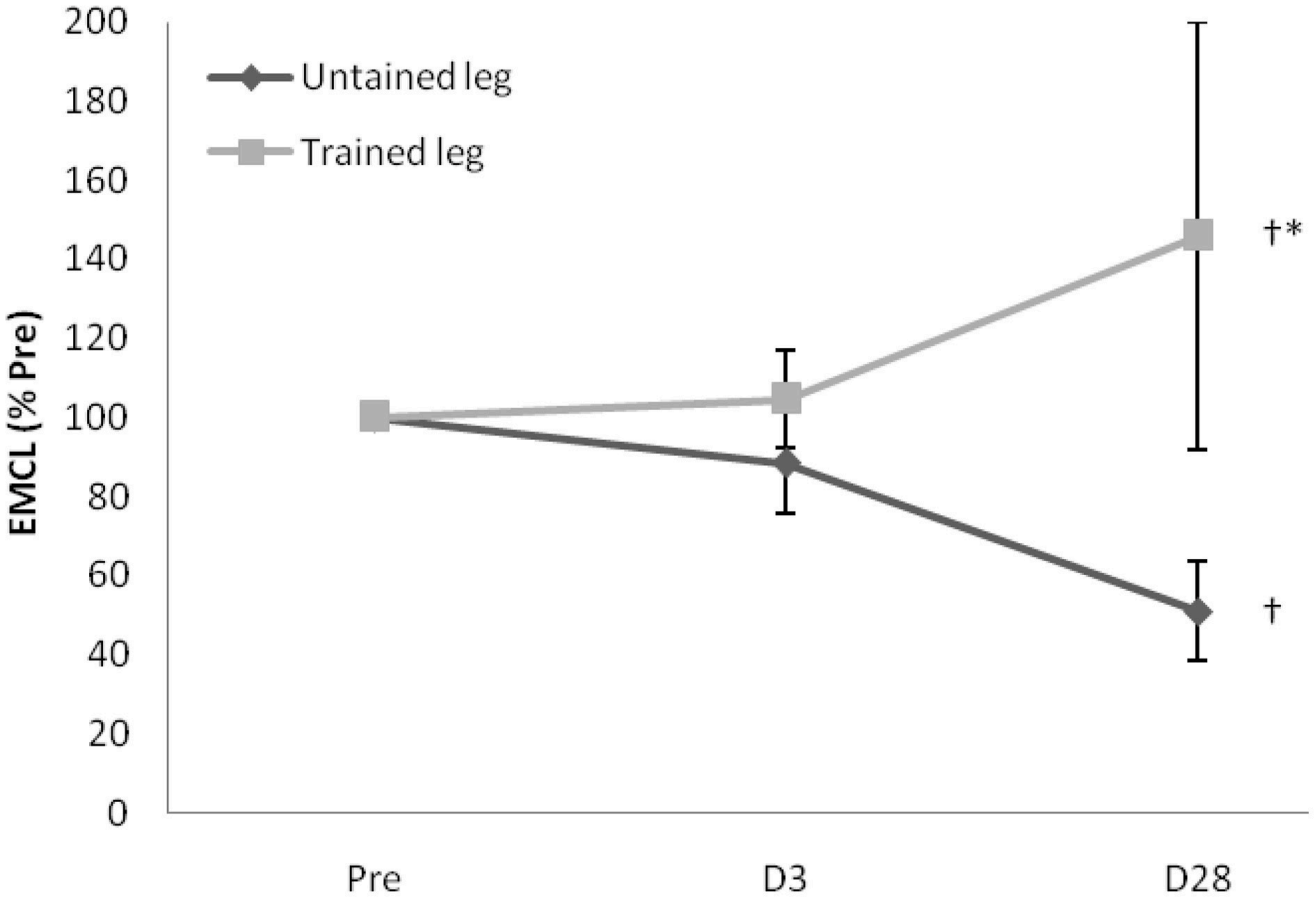
**Extramyocellular lipid (EMCL) contents of trained vastus lateralis continued to increase while untrained leg exhibited an opposing trend during the 28-day detraining period**. One unique participant had 39% and 417% increases at D3 and D28 above Pre, contributing the high average value and variation of trained leg. When two extreme data were replaced by mean, only a non-significant 11% increase is observed. ^†^Significance against Pre, *p* < 0.05. ^*^Significance against untrained leg, *p* < 0.05. D3 and D28: Day 3 and Day 28 after the last training bout.

**Figure 5:**
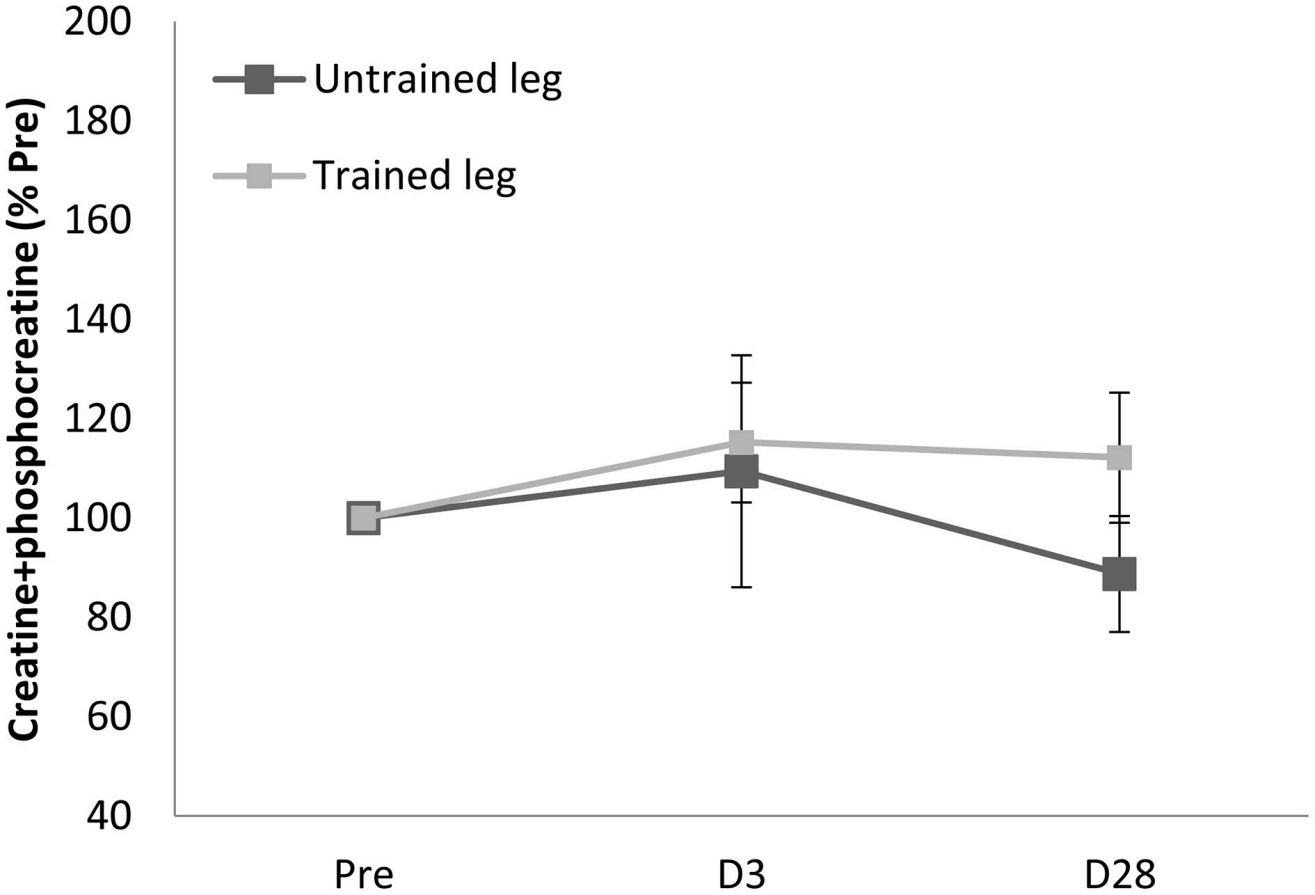
**Total creatine pool (creatine + phophocreatine) of vastus lateralis (B) was not significantly affected during entire observation period**. D3 and D28: Day 3 and Day 28 after the last training bout.

Proton MRS resonances for IMCL, EMCL, and total creatine are shown by Gaussian line shapes in the frequency domain (Figure 1A). A representative MR image for muscle is shown in Figure 1B. Three days after the last exercise training bout, muscle CSA of trained leg significantly increased (+20%) and untrained leg significantly increased to a lesser extent (+14%) above pre-trained level (Figure 2). CSA remained significantly greater than the pre-trained level after 28 days of detraining. Exercise training significantly increased IMCL of both trained and untrained vastus lateralis muscles (+77% and +79%, *P* < 0.05), observed 3 days after the last exercise training bout (Figure 3). However, IMCL reversed toward pre-exercise level in the previously untrained vastus lateralis muscle during 28 days of detraining (*P* > 0.05), but not as much change in the previously trained muscle. EMCL content of trained vastus lateralis was significantly increased (+46%, *P* < 0.05) while ECML content of untrained vastus lateralis was significantly decreased (−49%, *P* < 0.05) following 28 days of detraining (Figure 4). No significant difference was reached in total creatine content between trained and untrained vastus lateralis muscles (Figure 5).

## Discussion

In this study, we hypothesize that lipid storage (ECML and ICML) in a leg can be influenced by changing training activity of another leg. This is based on the previous observation from both animal and human studies showing a possible ectopic influence of lipid storage in a tissue. In animals, increasing muscle growth can decrease size of adipose tissues (McPherron and Lee, 2002; Diaz et al., 2012). In humans, triglyceride of skeletal muscle increases (Shaw et al., 2012) and thickness of subcutaneous (Krotkiewski et al., 1979) and abdominal fat (Vissers et al., 2013) decreases after exercise training. IMCL content of non-exercising human skeletal muscle has been found to increase after exercise training (Schrauwen-Hinderling et al., 2003). In this study, we asked the question whether EMCL and IMCL of untrained leg can be influenced during detraining of previously trained leg. The major findings of the study are: (1) a fall in EMCL in the untrained leg during detraining; (2) IMCL increases in both legs during training, but has only fallen in the untrained leg during detraining. The results of the study provide a support for the idea that lipid storage of an inactive tissue can be influenced by a changing metabolic demand of another tissue within the same human body.

The role of EMCL in muscle tissue is currently unclear. Unlike IMCL, EMCL of adipocyte is not consumed during muscular contractions (Brechtel et al., 2001). However, adipocytes in skeletal muscle are comparably closer to the working muscle fibers than abdominal adipocytes. Thus, EMCL of skeletal muscle tissue may serve as hydrocarbon source to stably maintain the increased basal demand on hydrocarbon sources after muscle tissue expansion.

In humans, lipids are used not only as fuel, but as the main source of acetyl-CoA, which provides the hydrocarbon backbone for synthesizing many biological molecules, such as cholesterol, ketones, phospholipids, some amino acids, and glycolipids. These biomaterials are required for constructing cells. Some of them can be exchanged across tissues via the circulation creating a whole-body hydrocarbon source redistribution and which is particularly in high demand when local cell turnover increases. Growth, normally occurring after destructive or damaging events, will attract hydrocarbon sources for fast cell repair and proliferation. It has been demonstrated that promoting either muscle size expansion (McPherron and Lee, 2002; Diaz et al., 2012) or tumor growth (Camus et al., 2014) can decrease fat mass effectively, suggesting that fatty hydrocarbon sources relocate toward the tissues with high cell damage and proliferation.

In this study, we do not provide evidence to explain the mechanism underlying asymmetrical changes of EMCL between legs. Cell death and renewal of muscle and adipose tissues are continuously taking place in human adults (Spalding et al., 2008). The balance of cell death and repopulation of a tissue determines the eventual size and morphology of local tissue. It would be interesting to see whether EMCL changes on both legs observed in the study is a result of asymmetrical local attraction and proliferation to stem cells in response to the one-leg challenge. In addition, differentiation of localized stem cell in skeletal muscle toward lineages of myogenesis or adipogenesis under different physical stressors such as exercise remains an unexplored field for the future.

In conclusion, decreased IMCL and EMCL levels in an untrained leg during a 4-week detraining suggest that the amount of lipid storage of inactive tissues can be influenced by energy-depleting experience of active tissues in the same human body. Our finding provides a support the ectopic influence on lipid storage by metabolic demand of distal tissue in the same human body.

## Funding

The study was funded by grants from University of Taipei and General Administration of Sport of China Project 2012B060.

### Conflict of interest statement

The authors declare that the research was conducted in the absence of any commercial or financial relationships that could be construed as a potential conflict of interest.

## Abbreviations

IMCL: intramyocellular lipid
EMCL: extramyocellular lipid
^1^H-MRS: proton magnetic resonance spectra
FIDs: free induction decays
TMA: trimethylamine
TCr: Total Creatine
Cr: Free Creatine
PCr: Phosphocreatine.
